# Treating myasthenia gravis beyond the eye clinic

**DOI:** 10.1038/s41433-024-03133-x

**Published:** 2024-05-24

**Authors:** Saiju Jacob

**Affiliations:** 1 University Hospitals Birmingham, Birmingham, UK; 2 Institute of Immunology and Immunotherapy, University of Birmingham, Birmingham, UK

**Keywords:** Neuromuscular disease, Diseases

## Abstract

Myasthenia gravis (MG) is one of the most well characterised autoimmune disorders affecting the neuromuscular junction with autoantibodies targeting the acetylcholine receptor (AChR) complex. The vast majority of patients present with ocular symptoms including double vision and ptosis, but may progress on to develop generalised fatiguable muscle weakness. Severe involvement of the bulbar muscles can lead to dysphagia, dysarthria and breathing difficulties which can progress to myasthenic crisis needing ventilatory support. Given the predominant ocular onset of the disease, it is important that ophthalmologists are aware of the differential diagnosis, investigations and management including evolving therapies. When the disease remains localised to the extraocular muscles (ocular MG) IgG1 and IgG3 antibodies against the AChR (including clustered AChR) are present in nearly 50% of patients. In generalised MG this is seen in nearly 90% patients. Other antibodies include those against muscle specific tyrosine kinase (MuSK) and lipoprotein receptor related protein 4 (LRP4). Even though decremental response on repetitive nerve stimulation is the most well recognised neurophysiological abnormality, single fibre electromyogram (SFEMG) in experienced hands is the most sensitive test which helps in the diagnosis. Initial treatment should be using cholinesterase inhibitors and then proceeding to immunosuppression using corticosteroids and steroid sparing drugs. Patients requiring bulbar muscle support may need rescue therapies including plasma exchange and intravenous immunoglobulin (IVIg). Newer therapeutic targets include those against the B lymphocytes, complement system, neonatal Fc receptors (FcRn) and various other elements of the immune system.

## Abstract (zh)

重症肌无力(MG)是影响神经肌肉接头的最典型的自身免疫性疾病之一, 自身抗体以乙酰胆碱受体(AChR)复合体为靶点。绝大多数患者的眼部症状包括复视和上睑下垂, 但可能会发展为全身性疲劳性肌无力。严重的延髓肌受累可导致吞咽困难、构音障碍和呼吸困难, 进而发展为需呼吸机支持的肌无力危象。鉴于该病以眼部起病为主, 眼科医生必须掌握鉴别诊断、调查和对其的管理, 以及不断更新的治疗方法。当病情仍局限于眼外肌(眼MG)时, 近50%的患者存在抗AChR(包括簇状AChR)的IgG1和IgG3抗体。在全身性MG中, 近90%的患者存在以上情况。其他抗体包括针对肌肉特异性酪氨酸激酶(Musk)和脂蛋白受体相关蛋白4(LRP4)的抗体。尽管对重复神经刺激的反应减弱是最常见的神经生理学异常, 但对有经验的医生来说, 单纤维肌电(SFEMG)是最敏感的有助于诊断的检查。最初的治疗应使用胆碱酯酶抑制剂, 随后使用皮质类固醇和类固醇保留药物进行免疫抑制治疗。需延髓肌支持的患者可能需抢救治疗, 包括血浆置换和静脉注射免疫球蛋白(IVIg)。新的治疗靶点包括针对B淋巴细胞、补体系统、新生儿Fc受体(FcRN)和免疫系统其它靶点的治疗。

## Introduction

Myasthenia gravis (MG) is the most common autoimmune disease affecting the neuromuscular junction (NMJ) clinically characterised by fatigable weakness of the ocular, limb and bulbar (speech, swallowing and respiratory) muscles. The incidence of MG varies between 1.7 to 21.3 per million person-years. The estimated United Kingdom (UK) prevalence of MG is 15 per 100,000 population [1, 2]. There is significant social, economic and emotional burden for patients with MG, which can often be more troublesome than the disabling symptoms and hospitalisations [3].

Most patients with MG present with ocular symptoms (mainly ptosis and ophthalmoplegia) and in ~20% of patients the disease remain localised to the eye muscles (ocular MG). In the remaining patients the disease can cause generalised symptoms which can range from being mild fatigue to those needing ventilatory support for respiratory crisis. The most commonly described antibodies are against the acetylcholine receptor (AChR) or muscle specific tyrosine kinase (MuSK).

## Pathophysiology at the neuromuscular junction

In the NMJ, nerve action potential provokes the activation of presynaptic voltage gated calcium channels (VGCC) which initiates a cascade of events eventually causing the release of acetylcholine (ACh) into the synaptic cleft. Acetylcholine binds to its receptor (AChR) in the synaptic folds of the postsynaptic membrane thereby opening the central pore of the receptors and subsequently the voltage gated sodium channels (VGSC), which then initiate the muscle action potential [4]. Normal NMJ function requires clustering of AChR in the crests of the synaptic folds which is achieved by a tyrosine kinase enzyme, MuSK. Activation of MuSK occurs during the development of NMJ by the interaction of agrin released from the developing motor axons with a post-synaptic protein, lipoprotein receptor related protein 4 (LRP4). Binding of this complex is crucial for the dimerisation and activation of MuSK. MuSK activation induces a series of phosphorylation reactions recruiting DOK7 and rapsyn and finally inducing clustering and stabilisation of AChR [5].

**Fig. 1:**
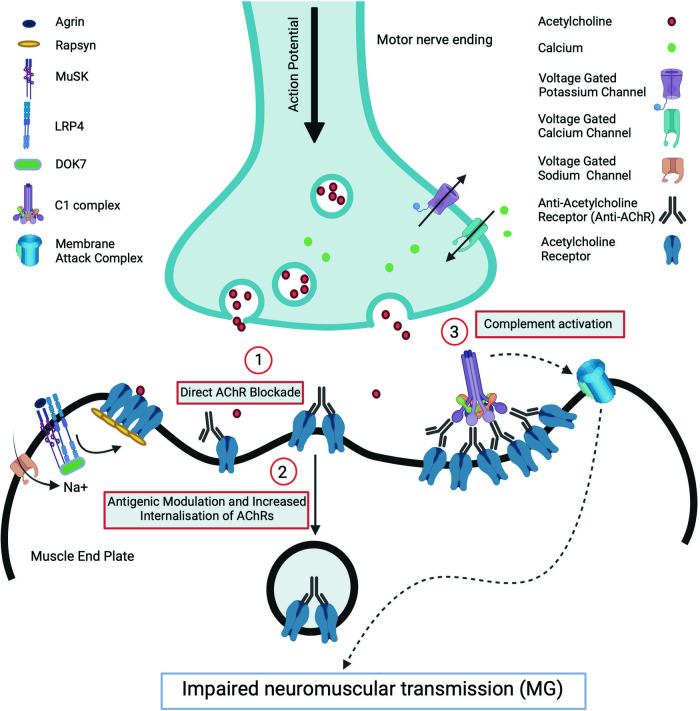
Neuromuscular transmission and immunopathogenesis of myasthenic disorders. The action potential arriving at the pre-synaptic terminal opens voltage gated calcium channels (VGCC) triggering release of Agrin and Acetylcholine receptor (AChR) to the synaptic cleft. The binding of ACh to its receptor (AChR) opens voltage gated sodium channels leading to muscle contraction. The clustering of AChRs at the neuromuscular junction (NMJ) is promoted by Agrin binding to the MuSK-LRP4 complex. The three main mechanisms by which AChR antibody causes neuromuscular damage is by: Antigenic modulation where Anti-AChR cross links AChRs, increasing the internalisation of AChRs (1), Direct blockade when Anti-AChR blocks the ligand binding site of Acetylcholine to AChR (2) and Complement activation (3). Anti-AChR-AChR complex activates the complement system, leading to the destruction of muscle end plate by Membrane Attack Complex. Other NMJ syndromes include Anti-MuSK MG (Anti-MuSK binds MuSK-Lipoprotein Receptor Related Protein 4 (MuSK-LRP4) and interferes with interaction of MuSK with other NMJ molecules and reduces AChR clustering) and LEMS (anti-VGCC binds VGCC at motor nerve terminal, blocking the calcium influx and calcium driven AChR vesicle release into NMJ).

The postsynaptic neuromuscular dysfunction in MG is produced by IgG autoantibodies which act by three main mechanisms: (1) direct functional blockade of the receptors, (2) antigenic modulation leading to receptor internalisation and degradation and (3) complement activation and destruction of the membrane [6]. AChR antibodies are predominantly of the complement fixing IgG1 and IgG3 subtypes. Complement fixation and generation of the membrane attack complex result in damage to AChR and voltage gated sodium channels in the post-synaptic membrane which reduces the safety factor and increases the threshold for excitation (Fig. 1). MuSK antibodies which are the IgG4 isotype do not significantly activate the complement pathway. Both IgG4 MuSK and IgG1/G2 LRP4 antibodies reduces neuromuscular transmission by inhibiting the clustering of AChR in the post-synaptic membrane [7].

## Role of thymus in MG

All patients with suspected MG should undergo imaging of the thymus gland. Combination of genetic susceptibility and environmental triggers such as viral infections have been implicated in the origin of MG leading to intra-thymic changes which play a pivotal role in the pathogenesis of AChR-MG [8]. The most accepted hypothesis centres around the failure of self-tolerance which occurs inside the thymus in AChR-MG. Self-tolerance is a balance between immune cell generation and the removal of auto-reactive lymphocytes in a timely fashion. During the T-cell maturation and differentiation in the thymus, the T regulatory cells (Tregs) are exposed to thymic myoid cells which express AChR and autoimmune regulatory (AIRE) medullary epithelial cells. The latter play an important role in the clonal deletion of T cells sensitised to auto-antigens during the development of central tolerance. The lymphocytes which display auto-reactivity are removed within the thymus whereas those that escape this culling are suppressed in the periphery by the Treg cells [9].

For each of the antibody subtypes of MG distinctive clinical phenotypes have been described [10]. There are three subtypes for AChR antibody positive MG: early onset, EOMG (<50 years of age), late onset, LOMG (>50 years) and thymoma associated MG, TAMG. EOMG predominantly occurs in women and have strong associations with human leucocyte antigen (HLA) DR3-D8 and thymic follicular hyperplasia. In contrast, LOMG often occurs in males, has no HLA association and may display anti-striational antibodies even though the thymus can be atrophic [11]. Up to 30% of MG have associated thymoma and often have co-existing anti-ryanodine receptor and anti-titin antibodies [12]. MuSK-MG tends to present with a bulbar-dominant phenotype and thymus is usually normal [13].

In EOMG, the germinal centres in the hyperplastic thymic follicles are implicated as the site of origin of autoimmunity. The mechanisms include aberrant production of cytokines and imbalance in the function of T effector and Treg cells. There is increase in the pro-inflammatory Th17 and T follicular helper cells which promote B cell activation and generate autoantibodies [9, 14]. In addition, the regulatory B cells which suppress autoimmunity are also functionally abnormal. In TAMG, pathological abnormalities consistent with immune proliferation in the areas adjacent to the tumour have been reported without any follicular hyperplasia. Thymomas express loss of AIRE making the cells susceptible for mounting autoimmune responses. LOMG on the other hand is associated with involuted thymus with paucity of the myoid cells and regulatory thymic cells which prevent autoimmunity. Thymus is usually normal and may not play a pathogenetic role in MuSK-MG [11].

## Ocular myasthenia gravis

Clinically, ptosis (droopy eyelids) may be unilateral or bilateral, is usually asymmetric, and patients often complain of diplopia (double vision) [15]. Ocular MG typically has wide variation in its severity and patients may also complain of dizziness, unsteady gait or blurred vision [16]. On examination, the less affected lid may be hyper-retracted in concordance with Hering’s law of equal innervation. One of the commonly observed signs, which is seen fairly commonly in ocular MG, is the drooping of the contralateral lid with passive elevation of a ptotic lid. When the patient looks down for 15 s and then rapidly looks up, the ptotic eyelid overshoots and then slowly droops to the previous ptotic position. This sign, named the Cogan’s lid twitch sign, is due to transient improvement of lid strength after resting of the levator palpebrae superioris muscle. Another commonly used technique is the Ice pack test where ice is filled in a thin surgical glove and kept against the closed eyelid of a seated patient for 2–5 min. The sensitivity of a 5-min icepack application ranged from 76.9% (when assessing the changes in strabismus deviation) to 92.3% (testing degree of ptosis as quantified by the change in marginal reflex distance, MRD) with a very high specificity (98.3%) [17]. The common bedside tests used to diagnose ocular myasthenia are shown in Box 1. Weakness of the extraocular muscles (ophthalmoparesis) in combination with ptosis and normal pupillary responses is highly suggestive of ocular MG. The main differential diagnoses of ocular myasthenia are mentioned in Table 1.

**Table 1:** Differential diagnosis of ocular myasthenia.

Differential diagnosis	Typical clinical features	Investigations
Cranial nerve palsies	Ophthalmoplegia will be restricted to the muscles supplied by the corresponding nerve (3, 4 or 6). Mydriasis with 3rd nerve palsy.	MRI and MRA of the brain
Multiple sclerosis	Internuclear ophthalmoplegia; optic neuritis; pyramidal and sensory signs	MRI brain—T2/FLAIR hyperintensities usually in the periventricular white matter with dissemination in space and time. Post-contrast images may demonstrate new and old lesions confirming dissemination in time, especially if there is only a single clinical event.
Chronic progressive external ophthalmoplegia	Progressive ptosis and slowly progressive symmetric extraocular muscle weakness	Mitochondrial genetics and muscle biopsy
Miller Fisher syndrome	Ophthalmoplegia with ataxia and areflexia	Ganglioside antibodies; EMG
Congenital myasthenia	Early onset	Genetic testing
Horner’s syndrome	Ptosis, miosis, anhidrosis, enophthalmos (normal eye movements)	Apraclonidine testing
Thyrotoxicosis	Proptosis, eye lid retraction	Thyroid function
Oculopharyngeal muscular dystrophy	Insidious onset and slowly progressive ptosis, ophthalmoplegia and dysphagia; family history	Genetic testing
Lambert Eaton myasthenic syndrome	Proximal muscle weakness with reduced reflexes; autonomic dysfunction	VGCC antibodies; EMG
Organophosphate poisoning	Fasciculations, excessive secretions, pinpoint pupils, typical odour, incontinence	Atropine therapeutic trial, plasma and red cell cholinesterase levels
Botulism	Oculobulbar weakness with descending paralysis	Mouse bio-assay using serum, gastric secretions, stool or food samples; ELISA, PCR

*ELISA* enzyme linked immunosorbent assay, *EMG* electromyogram, *MRA* magnetic resonance angiogram, *MRI* magnetic resonance imaging, *PCR* polymerase chain reaction, *VGCC* voltage gated calcium channel.

Box 1 Common bed-side tests to diagnose ocular myasthenia

1. Assess fatiguability
  Ask patient to hold an upgaze for 2–3 min and assess fatigue of the levator palpebare superioris, by assessing the change in the interpalpebral fissure distance or the marginal reflex distance, MRD.
2. Ice Pack test
  Although a commercial ice pack can be used, practically this is done by holding an ice-filled thin surgical glove over the closed eye for 2 min. When assessing ophthalmoplegia, this may need to be kept over the eye for up to 10 min. A change in the interpalpebral distance or marginal reflex distance of $\ge$ 2 mm is considered to be a positive response. Improvement in ophthalmoplegia is considered significant when there is a change in prism dioptre (PD) by 50% or at least 10 PD, when pre-test deviation is more than 20 PD. Test is unlikely to be positive in Horner’s syndrome and cranial nerve palsies and can be positive even in myasthenia which is co-existent thyroid eye disease.
3. Cogan’s lid twitch sign
  This is elicited when there is visible ptosis. Patient is asked to look down for 15 s followed by changing to primary gaze (looking straight ahead) and observing a twitch in the droopy lid.
4. Tensilon test
  Historically injection of short acting cholinesterase inhibitors like edrophonium was used to assess the change in ptosis or ophthalmoplegia, but this is very rarely used nowadays due to safety concerns.

### Why are ocular muscles more susceptible to MG weakness?

Several reasons have been suggested for the predilection of extraocular muscles (EOM) in MG [18, 19]:

1. EOM twitch fibres have higher firing frequencies, lower density of AChR and less number of packets of ACh released, thereby increasing their susceptibility to fatigue.
2. 20% of EOM contain multi-terminal fibres, which have sparse junctional folds, lower AChR density and do not have a safety factor.
3. There have been suggestions that antibodies in ocular MG patients’ serum react more strongly with ocular muscle antigens [20, 21], although further studies have refuted this argument [22]. It is likely that the previous findings were due to the high proportion of normal adult AChRs in ocular muscles compared to fetal AChRs in denervated leg muscles. EOMs (but probably not the levator muscles) have been thought to contain both the fetal (γ) and adult (ε) form of AChRs as opposed to the extremity muscles which contain exclusive adult isoforms [23]. Ocular MG patients’ serum reacts more strongly with adult than fetal AChRs [24].
4. EOM differentially express immune-related genes making them more susceptible to autoimmune diseases [25].
5. Diminished complement regulatory activity could render the EOM more susceptible to autoimmune attack [26].
6. Lastly, but perhaps most importantly, minor weakness of EOM can misalign the visual axis to produce symptoms. EOM may be disproportionately sensitive to the disease. Indeed, many ocular MG patients show subclinical neurophysiological defects in the peripheral limb muscles.

Previously, an animal model for ocular MG has been developed using mice transgenic for HLA-DQ8 and HLA-DR3 with deficient MHC-Class II after immunising with a recombinant AChR α-subunit [27]. It is not clear why this model should develop ocular signs and careful comparisons with other models have not been done. These and other similar models could prove a valuable tool in studying the pathophysiological mechanisms underlying ocular MG.

## Antibodies in MG

Autoantibodies are seen in ~50% of patients with ocular MG [28] whereas nearly 95% of generalised MG patients have antibodies against various components of the post-synaptic membrane [10]. 85% of antibodies are targeted against the AChR and antibodies against other parts of the NMJ, namely MuSK, LRP4 and agrin have been reported in 5%, 2% and less than 1% respectively (Fig. 1). This leaves ~5–7% of patients where a specific antibody cannot be identified using the currently available assays and these are referred to have seronegative MG [11]. As mentioned earlier, in the pure ocular MG the seronegative status can be closer to 50%. Antibodies against several other molecules have been described including acetylcholinesterase, ColQ, titin, ryanodine, Kv1.4 and cortactin, but their exact pathophysiological role is unknown [29, 30].

## Neurophysiology in MG

The two most commonly employed techniques for the diagnosis of ocular MG are repetitive nerve stimulation (RNS) and single fibre EMG (SFEMG) studies. Although the diagnostic yield of RNS is increased when the orbicularis oculi, orbicularis oris or nasalis muscles are tested, patients often find these examinations difficult to tolerate. Less than 50% of ocular MG patients demonstrate decremental response as opposed to 75% of generalised MG patients [16]. SFEMG has been described as the most sensitive test to identify a defect in neuromuscular transmission, especially when done by experienced neurophysiologists [31–33].

SFEMG has been found to be a reliable and sensitive technique for the diagnosis of neuromuscular transmission failure [32, 34, 35]. In SFEMG, the action potentials from two adjacent muscle fibres arrive at the recording electrode at varying time intervals. This represents the combined jitter at the two end-plates. The standard deviation of these variations can be used to express jitter. In order to assess the status of neuromuscular transmission, usually 20 potential pairs are examined [32]. Increased jitter is a sign of impaired neuromuscular transmission which can be sub-clinical (i.e. without apparent weakness) in the early stages, but can lead to blockage of impulse transmission (also called jitter blocking), which is always associated with clinical weakness. In other words, a normal jitter in an apparently weak muscle (e.g.: ptosis) will be highly unlikely to be due to myasthenia gravis or another neuromuscular transmission disorder.

## Management of myasthenia gravis

The therapeutic strategy in MG is guided by the clinical pattern (ocular versus generalised), autoantibody subtype, and disease severity. Current therapy of MG revolves around symptomatic treatment (cholinesterase inhibitors), long term steroids and steroid-sparing immunomodulation including thymectomy and rescue therapy in acute crisis. A summary of the various pharmacological therapies currently used in MG (including usual dosing) is shown in Table 2.

**Table 2:** Commonly used medications in MG.

Medication	Usual adult dose	Common side effects	Caution/Monitoring	Notes
Pyridostigmine	Start at 30 mg TDS- QDS, slowly titrate by 30 mg every 2–3 days up to 360 mg/day; can rarely go up to 480 mg/day	Muscle/abdominal cramps, diarrhoea, increased salivation, bladder urgency	Bradycardia, asthma, heart disease	Propantheline 15 mg tds-qds may be used to counteract cholinergic side effects like cramps and diarrhoea (can use up to 30 mg qds)—best taken 30 min before each Pyridostigmine dose. If Propantheline unavailable, potential options include Hyoscine (Buscopan®) or Mebeverine.
Prednisolone	Start at 5 mg OD increased by 5 mg every 1–2 weeks until on 30 mg OD (0.5 mg/kg/day—ocular MG) or 60 mg OD (1 mg/kg/day—generalised MG) or until significant improvement, whichever is earlier.	Short term: heart burn, sleep disturbance, mood changes, acne, weight gain, blurred vision. Long term: gastric ulcers, osteoporosis, proximal myopathy, fluid retention, weight gain, diabetes mellitus, hypertension and infection risk	Avoid leaving patients at high doses for long periods. Prescribe stomach (Omeprazole/Lansoprazole) and bone protection (Calcium/Vitamin D and Alendronic acid) tablets. Periodic bone density scans, regular blood sugar, HbA1C and blood pressure monitoring.	Continue effective dose for 4 weeks; then reduce by 5 mg every 2 weeks until on 30 mg OD and thereafter by 5 mg every 4 weeks until on 15 mg OD. Further reductions in steps of 2 mg every 4 weeks until on 9 mg OD and then cautiously by 1 mg every 4 weeks to the lowest possible maintenance dose
Azathioprine	Start at 75 mg OD (1 mg/kg) and titrate over 2–3 months to reach 2.5 mg/kg/day.	Nausea, vomiting, myelosuppression, liver dysfunction, risk of skin cancer	Always check and ensure TPMT levels normal, before initiation. Check FBC, U&E and LFT every 2–4 weeks for 3 months and three-monthly thereafter	Lymphopenia and macrocytosis are common and often desirable. Drug of choice in women of childbearing age
Mycophenolate mofetil	Start at 500 mg BD, increased within 4 weeks to 1000 mg BD	Dyspepsia and myelosuppression	Check FBC, U&E and LFT every 2–4 weeks for 3 months and three-monthly thereafter	Avoid, if risk of pregnancy
Methotrexate	Start at 7.5 mg/week, increase up to 20 mg/week as per response and tolerance	Nausea, vomiting, mouth ulcers, myelosuppression, respiratory and hepatic dysfunction.	Chest X-ray prior to starting. Check FBC, U&E and LFT every 2–4 weeks for 3 months and three-monthly thereafter	Folic acid to be given 5 mg/6 days a week except on the day of Methotrexate
Ciclosporin	Start at 25 mg BD increased by 50 mg every 3–7 days until on 2.5 mg/kg/day	Nausea, vomiting, excessive body hair, renal failure, high blood pressure	Regular monitoring of blood pressure and renal function	Can monitor drug levels
Tacrolimus	50 mg/kg/day	Nausea, vomiting, myelosuppression, high blood pressure, diabetes, renal and hepatic dysfunction	Check FBC, U&E and LFT every 2–4 weeks for 3 months and three-monthly thereafter. Regular BP monitoring	Trough level to be maintained at 5–10 ng/ml
Cyclophosphamide	1–3 mg/kg/day	Nausea, vomiting, diarrhoea, fatigue, haemorrhagic cystitis, myelosuppression	Check FBC, U&E and LFT every 1–2 weeks for 3 months and monthly thereafter	Usually used in refractory disease
Rituximab	1 g/day IV infusion on D1 and D14	Infections; reactivation of TB, Hepatitis etc	Check FBC, U&E, LFT, Chest X-ray, Hepatitis serology before initiation. Monitor blood counts, immunoglobulins and CD19 levels during therapy	If CD19 levels rise to more than 1%, consider re-dosing if symptoms are coming back

*BD* twice daily, *CD19* cluster of differentiation 19 cells (B lymphocytes), *FBC* full blood count, *LFT* liver function tests, *OD* once daily, *QDS* four times a day, *TDS* three times a day, *U&E* urea and electrolytes.

With the advent of newer therapies, myasthenia patients are often systematically assessed in clinic using standardised scoring systems which include patient-reported outcomes like the Myasthenia Gravis Activities of Daily Living (MG-ADL) or Myasthenia Gravis Quality of Life (MG-QoL-15) scores. In addition, MG Composite, a combined patient-reported outcome and physician examination score is also relatively easy to perform. All of these scores are usually done regularly in many specialist myasthenia clinics. More detailed assessment can be done using Quantitative of Myasthenia Gravis (QMG) scale, which is more often done in a clinical trial setting along with the other scores. A detailed discussion of these scores is outside the remit of this article.

### Cholinesterase inhibitors

Cholinesterase inhibitors such as pyridostigmine (usual dose 30–60 mg three to four times a day, titrating up if needed up to 360 mg/day in divided doses) can produce rapid relief of symptoms in mild MG and are usually prescribed as the initial therapy. Patients may develop cholinergic side effects like abdominal and limb muscle cramps, diarrhoea etc and these can be counteracted by drugs like Propantheline. Some patients with MuSK-MG may show a paradoxical worsening with cholinesterase inhibitors.

### Corticosteroids

Majority of patients require suppression of autoantibody production with immunosuppressive treatment which remains the cornerstone of MG therapy. Oral corticosteroids are initially used to induce remission while long-term maintenance is achieved with either low dose oral corticosteroids or non-steroidal immunosuppressants. Even though highly effective, the beneficial effects have to be balanced with the morbidity associated with long term corticosteroid usage. Worsening of bulbar symptoms leading to myasthenic crisis can occasionally be precipitated by high dose steroids, although is less likely to occur with gradual titration of the dose with close monitoring.

### Steroid sparing immunosuppressants

Azathioprine, mycophenolate mofetil, methotrexate, ciclosporin and tacrolimus are the most frequently used non-steroidal agents. Agents like cyclophosphamide are usually reserved for poorly responsive patients. Azathioprine is probably the safest of these in women of childbearing age, but the onset of action is often delayed by several months (sometimes even up to 18 months). It is essential that levels of Thiopurine Methyl Transferase (TPMT) are checked before starting Azathioprine since TPMT deficiency leads to accumulation of active 6-mercaptopurine and its toxic metabolites, 6-thioguanine nucleotide analogues causing bone marrow suppression. For faster onset of action, many myasthenia specialists prefer Mycophenolate (especially where there is no teratogenic risk) but even this does not become effective for at least 3–6 months.

### Plasma exchange and intravenous immunoglobulins

Rapidly acting, but short-lasting agents such as therapeutic plasma exchange (PLEX) or intravenous immunoglobulin (IVIg) are used in patients with impending respiratory crisis and severe MG [36]. Even though there is limited randomised control trial evidence, short term benefit of PLEX in myasthenic crisis has been shown in several case series [37]. Both PLEX and IVIg have rapid onset of action but are generally considered unsuitable for long term maintenance therapy due to potential side effects [38, 39]. Nevertheless in treatment-refractory MG it is not unusual for patients to receive regular IVIg, PLEX or immunoadsorption [40, 41]. Subcutaneously administered immunoglobulin (SCIg) is an attractive alternative to IVIg providing not only more persistent blood levels and fewer systemic adverse effects, but also reducing health care resource utilisation, preserving patient autonomy and providing better quality of life [42, 43].

### Thymectomy

In all MG patients who have a thymoma or those who are AChR antibody positive, therapeutic thymectomy is recommended. The beneficial effect of thymectomy in older patients and antibody negative MG is not fully established, and it is generally recommended in onset of disease less than 50 years of age [36, 44, 45]. Practically however, it is often used until the age of 65 if the patients are otherwise fit and healthy. Thymectomy is not recommended in MuSK-MG since there are no consistent thymic abnormalities in this sub-group [46].

### Rituximab

Rituximab is a chimeric mouse/human antiCD20 mAb which rapidly depletes the mature and memory B cells in the peripheral blood while largely sparing the pre-B cells and plasma cells located in the bone marrow and secondary lymphoid organs. This effect is achieved by target cell apoptosis, antibody-dependent cell-mediated cytotoxicity and complement-dependent cytotoxicity, with the effect lasting up to 6 months [47].

Rituximab spares the long-lived plasma cells which do not express CD20 while it depletes the short-lived plasma cells and B regulatory cells (interleukin-10 producing cells) which have CD20 expression [48]. AChR antibodies are produced by the long-lived plasma cells whereas short-living plasma blasts are thought to be the source of the MuSK antibodies [49]. This might be the reason that some systematic reviews suggest lower corticosteroid requirements, better remission rates (47% vs. 16%) and minimal manifestation status (72% vs. 30%) in patients with MuSK-MG as opposed to AChR-MG [50, 51].

The standard Rituximab regimen includes two infusions of 1 g 2 weeks apart (some clinicians use 375 mg/m^2^ weekly infusions for 4 weeks). Reinfusions are usually guided by clinical symptoms or B cell repopulation [50]. Two systematic reviews showed unequivocal clinical improvement in 68–77% of AChR-MG patients [52, 53]. When routine and low dose Rituximab (171 and 89 subjects respectively) were compared in refractory AChR-MG no significant difference in the therapeutic outcomes or side effects were noted. Newer retrospective studies (albeit without a clear comparator arm) have shown good efficacy and safety profile using low dose Rituximab regimens as monotherapy or add-on immunotherapy in new-onset and early MG [54–56]. When used early (the mean time of initiation since MG diagnosis of 1.6 months with a median dose of 183 mg) Rituximab has been shown to produce sustained clinical remission and also discontinuation of steroids in nearly 80% of patients by 22 months [54, 56]. A recent double-blind placebo controlled trial used low dose Rituximab (single IV infusion of 500 mg) at diagnosis and has shown efficacy in lowering the MG scores at week 16 needing low (<10 mg) Prednisolone dose without any rescue therapies (71% vs. 29%, *p* = 0.007) [57].

Adverse effects have been documented in 4 to 26% of patients, most commonly infusion reactions, reduced blood cell counts, opportunistic infections and prolonged hypogammaglobulinemia [50, 53].

## Role of early fast acting therapies in MG

The traditional treatment plan in MG used to be to start low and titrate up slowly. This can cause significant morbidity from mainly steroid-related side effects affecting the quality of life. In recent years, increasing evidence is now gathering on the use of early and fast acting therapies in achieving minimal manifestation earlier and reducing the dose and duration of steroid use. In a large study looking at 700 patients, half of the group were treated with intravenous steroids, PLEX, IVIg or a combination of these within the first few days of diagnosis and there is significant (*p* < 0.0001) difference in the proportion of patients achieving minimal manifestations (74.3% vs. 58.9%), disease duration (8.6 vs. 14.2 years), maximum prednisolone dose (20.2 vs. 25.0 mg) and duration of use of Prednisolone dose >10 mg (2.0 vs. 3.5 years) when compared with the more “conventional” regimens [58].

## Newer immunotherapies in MG

**Fig. 2:**
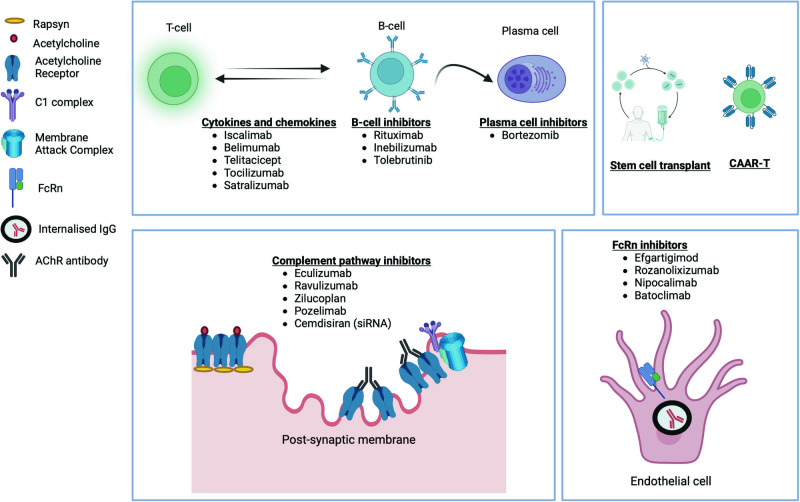
Newer therapeutic targets in myasthenia gravis. Several therapeutic targets are currently being studied in MG. This includes the complement pathway inhibitors turning off the membrane attack complex formation, FcRn inhibitors reducing the levels of circulating AChR antibodies, inhibition of B lymphocytes and plasma cells or targeting the cytokine pathways.

In the last few years, various novel treatment options are being studied in MG and these predominantly target various cells and immune pathways implicated in the pathogenesis of MG (Fig. 2) [59]. Inhibition of complement pathway and neonatal Fc receptor (FcRn) are two of the most successful mechanisms identified among them. Inhibition of B and T cells by direct depletion or through cytokines are also being studied. A few of the drugs, namely, Eculizumab, Ravulizumab, and Efgartigimod, have obtained approval from regulatory agencies while the majority are in various phases of development [60, 61]. A summary of the various newer therapeutic options is shown in Table 3 (adapted from [59]).

**Table 3:** New and emerging therapies in MG.

Drug	Mechanism of action	Sub-type of MG	Regimen	Notes and RCT evidence
Complement inhibitors				
Eculizumab	Recombinant humanised IgG2/4 monoclonal antibody against C5	AChR+ gMG	IV; Induction of 900 mg weekly for 4 weeks followed by 1200 mg maintenance every 2 weeks	Phase 3 RCT results: QMG: Eculizumab vs. Placebo = 54.7 vs. 70.7 (*p* = 0.0129); MG-QoL-15: Eculizumab vs. Placebo = 55.5 vs. 69.7 (*p* = 0.0281) (**REGAIN**, NCT01997229) U.S. FDA approved for treatment of adults with AChR+ gMG—Oct 2017
Ravulizumab	Long-acting recombinant humanised monoclonal antibody against C5	AChR+ gMG	IV; A single loading dose of 2400–3000 mg followed by maintenance doses of 3000–3600 mg every 8 weeks	Phase 3 RCT results: MG-ADL from baseline in treatment vs. placebo = −3.1 vs. −1.4 (*p* < 0.001) (**CHAMPION MG**, NCT03920293) U.S. FDA approved for treatment of adults with AChR+ gMG—Apr 2022
Zilucoplan	Macrocyclic peptide binding C5 and C5b	AChR+ gMG	SC; once daily dose of 0.3 mg/kg	Phase 3 study showed positive results. MG-ADL from baseline in treatment vs. placebo = least squares mean change −4.39 [95% CI –5.28 to –3.50] vs. −2.30 [–3.17 to –1.43]; least squares mean difference −2.09 [−3.24 to −0.95) (*p* = 0.0004); (**RAISE, NCT04115293**) U.S. FDA approved for adults with generalised AChR+ve gMG—Oct 2023
Pozelimab	Fully humanised IgG4 monoclonal antibody inhibiting C5 complement	AChR+ or LRP4+ gMG	SC; alone or in combination with Cemdisiran	Phase 3 study is ongoing (**NCT05070858**)
Cemdisiran	siRNA suppressing hepatic C5 synthesis	AChR+ or LRP4+ gMG	SC; alone or in combination with Pozelimab	Phase 3 study is ongoing (**NCT05070858**)
Gefurulimab (ALXN1720)	Anti-C5 humanised bi-specific VHH (variable domain on a heavy chain) antibody (nanobody)	AChR+ gMG	SC; weight-based dose once weekly	Phase 3 study is ongoing (**NCT05556096**)
Danicopan (ALXN2050)	Small molecule complement pathway factor D inhibitor	AChR+ gMG	Oral; 120 mg or 180 mg	Phase 2 study is ongoing (**NCT05218096**)
FcRn blockers				
Efgartigimod	FcRn antagonist	All antibody subtypes of MG	IV, first cycle of 10 mg/kg/dose weekly for 4 weeks and repeat cycles at variable intervals based on symptom recurrence	U.S. FDA approved for treatment of AChR+ gMG in adults—Dec 2021 (Used in the UK by an Early Access to Medicine Scheme since 2022)
Rozanolixizumab	Anti-FcRn monoclonal antibody	All antibody subtypes of MG	SC, in phase 2 study 7 mg/kg was given weekly for 3 weeks followed by weekly 7 mg/kg or 4 mg/kg injections for 3 more doses	Phase 3 study has been completed (**NCT03971422**)
Nipocalimab (M281)	Anti-FcRn monoclonal antibody	All antibody subtypes of MG	IV infusion of 60 mg/kg every 2 weeks, multiple doses experimented in phase 2 trial	Phase 3 study is ongoing (**NCT04951622**)
Batoclimab (RVT 1401)	Anti-FcRn monoclonal antibody	All antibody subtypes of MG	SC 340 or 680 mg every week induction followed by maintenance of 340 mg every 1 or 2 weeks	Phase 3 study is ongoing (**NCT05403541**)
ABY-039	Bivalent antibody-mimetic against FcRn	–	–	Phase 1 study was prematurely terminated
B-cell and plasma cell therapies				
Rituximab	Anti CD20 monoclonal antibody	All antibody subtypes of MG	IV infusion. Various regimens are used, commonly two doses of 1 g 2 weeks apart or 375 mg/m^2^ infusions repeated weekly for 4 weeks	One phase 2 study was completed, extensive class IV data
Inebilizumab	Anti CD19 monoclonal antibody	AChR+ or MuSK+ MG	IV Infusion, 300 mg two doses 2 weeks apart and maintenance after 6 months	Phase 3 study is underway (**NCT04524273**)
Obinutuzumab	Anti CD20 monoclonal antibody	–	IV infusion as 6 treatment cycles, Dose of 100 mg in day 1, 900 mg in day 2, 1000 mg in days 8 and 15 of the first cycle followed by 1000 mg on the first day of the subsequent cycles	No ongoing study, anecdotal evidence emanates from a single case report
Bortezomib	26S proteasome inhibitor	All antibody subtypes of MG	SC, administered in 2 treatment cycles, each with 4 injections of 1.3 mg/m^2^ body surface per cycle	Phase 2 study was terminated due to recruitment difficulties
Tolebrutinib	Bruton’s tyrosine kinase inhibitor	All antibody subtypes of MG	Oral once daily dose	Phase 3 study is underway (**NCT05132569**)
TAK-079	Anti CD38 monoclonal antibody	AChR+ or MuSK+ MG	SC injection weekly with one of 2 doses for 8 weeks	Recruitment has been completed for phase 2 study (**NCT04159805**)
Chemokine and cytokine pathway targeting therapies				
Belimumab	Monoclonal antibody against BAFF	AChR+ or MuSK+ MG	IV 10 mg/kg every 2 weeks for 4 weeks and then once every 4 weeks	Phase 2 study has been completed
Telitacicept (RC18)	Inhibition of BAFF and APRIL	AChR+ or MuSK+ MG	SC 160 mg or 240 mg weekly	Phase 2 study is ongoing (**NCT04302103**)
Iscalimab (CFZ533)	Anti CD40 monoclonal antibody	All antibody subtypes of MG	IV infusion 10 mg/kg, 2 to 4 weekly doses	Phase 2 study has been completed
Tocilizumab	IL-6 receptor antibody	AChR+ MG	IV infusion every 4 weeks	Phase 2 study is ongoing (**NCT05067348**)
Satralizumab	IL-6 receptor antibody	All antibody subtypes of MG	SC, 120 mg every 2 weeks for 4 weeks and every 4-weeks maintenance thereafter	Phase 3 study is ongoing (**NCT04963270**)
Tofacitinib	Intracellular signalling disruption by inhibition of Janus kinases	AChR+ or seronegative MG	Oral, 5 mg twice a day	Early phase 1 study is underway (**NCT04431895**)
Miscellaneous				
Subcutaneous immunoglobulin	Broad spectrum immunomodulator	AChR+, MUSK+ or LRP4+ MG	1–2 g/kg over 4 weeks, equivalent to IVIg doses	Prospective open label trial has been completed. Phase 2 study is underway (**NCT04728425**)
CAR T cell therapy	Targeted depletion of autoimmune cells with genetically modified T cells	All antibody subtypes of MG	IV infusion	Phase 2 study is ongoing (**NCT04146051**)
CAAR T cell therapy	Targeted depletion of autoantibody- expressing immune cell clones with genetically modified T cells	AChR+ or MuSK+ MG	IV infusion, multiple dosage regimens	Phase 1 open label study for MuSK MG is underway (**NCT05451212**)
Autologous HSCT	Immune ablation and repopulation	All antibody subtypes of MG	–	No ongoing trials, class IV data

*APRIL* a proliferation-inducing ligand, *AChR+* acetyl choline receptor antibody positive, *BAFF* B-cell activating factor, *CAAR* chimeric autoantibody receptor, *CAR* Chimeric antigen receptor, *FcRn* neonatal Fc receptor, *HSCT* hematopoietic stem cell transplantation, *IL-6* interleukin 6, *IV* intravenous, *IVIg* intravenous immunoglobulin, *LRP4+* low density lipoprotein receptor-related protein 4 antibody positive, *MG* myasthenia gravis, *MuSK+* muscle specific kinase antibody positive, *SC* subcutaneous, *siRNA* small interfering ribonucleic acid.

### Complement pathway inhibitors

**Fig. 3:**
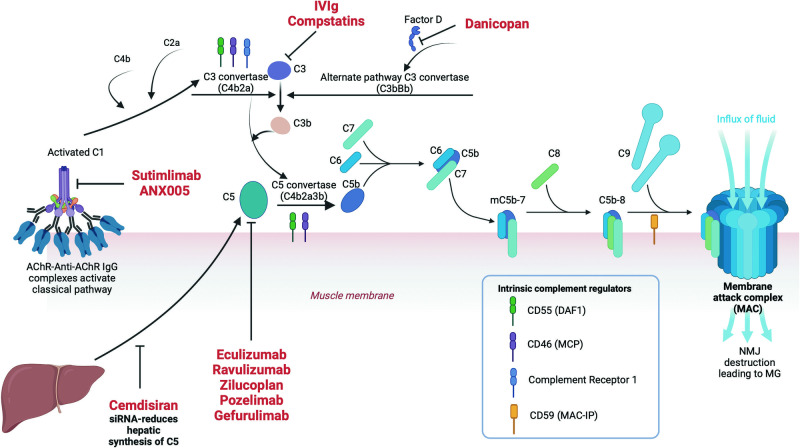
Components of the complement pathway and emerging therapies in MG. The classical complement pathway is activated by the binding of AChR antibody to its target antigen which leads to cascade of reactions culminating in the formation of a membrane attack complex and thereby damaging the neuromuscular junction. Several molecules are currently being used or studied in MG which affects different parts of this pathway.

An overview of the complement system and the potential therapeutic targets are shown in Fig. 3. In the classical complement pathway, antigen-antibody complexes bind to the six globular heads of C1q, activating C2 and C4 to form C3 convertase (C2a4b), which converts C3 to the active C3b. C3b cleaves C5 to its active form C5b, which in turn forms the membrane attack complex (MAC) with C6, C7, C8 and C9. The MAC forms lytic pores on the cell surface leading to cell destruction and thereby neuromuscular transmission failure.

There are various direct and indirect evidence for the role of complement in the pathogenesis of MG:

1. NMJs from myasthenia gravis patients and experimental models of myasthenia show deposition of C3 fragments and membrane attack complex (MAC) [62].
2. Depletion of complement protects animals from experimental myasthenia [63].
3. Experimental myasthenia can be prevented by anti-complement component C6 [64] or by soluble complement receptor 1 (sCR1), a complement inhibitor [65].
4. C5 gene influences the development of murine myasthenia gravis [66].
5. Mice lacking the complement regulator gene, decay accelerating factor (DAF), are more susceptible to experimental myasthenia [67].
6. Knocking out two complement regulator genes, DAF and CD59, produces severe respiratory weakness in an active immunisation model of AChR-myasthenia [68, 69].

Complement cascade can be disrupted by various molecules with the prime targets being C5, along with components of C3 and C1 [70]. C5 inhibitors like Eculizumab, Ravulizumab, and Zilucoplan have shown to be the most beneficial in clinical trials while more studies with other molecules are in the pipeline [71].

#### Eculizumab

Eculizumab is a humanised monoclonal antibody (mAb) directed against the C5 component of the complement pathway. Eculizumab prevents the C5 cleavage to C5a and C5b and thus inhibits the C5a-induced chemotaxis of inflammatory cells and the formation of MAC. Eculizumab has been used in paroxysmal nocturnal haemoglobinuria (PNH) [72] and atypical haemolytic uraemic syndrome (HUS) [73] and more recently for aquaporin IgG positive neuromyelitis optica spectrum disorder (NMOSD) [74].

Eculizumab has been found to be safe and effective in MG in a placebo-controlled randomised clinical trial (REGAIN). Even though the study failed to attain significance for the primary endpoint (mean rank of 56.6 vs. 68.3, *p* = 0.0698), the pre-specified secondary outcomes (changes in QMG and MGQOL-15 scores and the responder analysis for MG-ADL and QMG) were significantly better in the Eculizumab group starting from week 1 and sustained through week 26, and fewer patients in the active group needed rescue therapy [75]. In a post hoc analysis, 25% of refractory MG on Eculizumab had attained minimal manifestations status at 26 weeks, which was double that of the placebo group [76]. The side effects were mild to moderate, including headache, upper respiratory infection and nasopharyngitis, with no difference between the groups. No patients developed meningococcal infection.

The open label extension (OLE) phase of the Eculizumab study showed a reduction of 75% in the episodes of myasthenic worsening compared to the baseline and rapid improvements in all the myasthenia specific scores. No safety issues were reported [77]. At the end of the OLE, 84.7% showed clinically meaningful improvement in MG-ADL scores (≥3 points) and 71.4% patients showed improvement in QMG scores (≥5 points). A significantly higher proportion of Eculizumab-treated patients attained minimal symptom expression (defined as MG-ADL of 0–1 or MG-QoL15 score of 0–3) at week 26 of REGAIN [78]. In addition, Eculizumab has been shown to be beneficial in patients who were previously receiving chronic IVIg [79], Rituximab [80] or were ventilator-dependent [81].

The clinical trials, various subgroup studies and case series have clearly established the role of Eculizumab as a rescue therapy in refractory MG, but its role as a first-line agent and duration of therapy are undefined. Even though it was licensed to use in MG in 2019, the annual cost of therapy which exceeds half a million US dollars has been a major deterrent to the wider use of this drug [82, 83].

#### Ravulizumab

Ravulizumab is a long-acting C5 complement inhibitor with a mechanism of action similar to eculizumab, which only needs fewer infusions. In the phase 3 randomised placebo-controlled CHAMPION-MG study (NCT03920293), 175 adults with symptomatic AChR antibody positive gMG were recruited to receive ravulizumab infusion versus placebo. The primary efficacy endpoint of significant improvement in MG-ADL and for the secondary outcomes were achieved in the treatment group at 26 weeks, without any significant side effects [84].

#### Zilucoplan

Zilucoplan is a small macrocyclic peptide molecule which prevents the terminal activation of the complement cascade by binding to C5 complement component. It also binds to the existing C5b to prevent its attachment to C6. The advantages of this molecule include its small size which ensures good NMJ penetration, ability to concomitantly administer IVIg therapy since this is not an antibody and the potential for self-administration (given the once daily subcutaneous route of delivery) [85].

#### Pozelimab with Cemdisiran

Pozelimab is a human mAb which acts on the C5 complement whereas Cemdisiran is a small synthetic interfering ribonucleic acid (siRNA) which can suppresses the production of C5 in the liver. Both molecules are given as subcutaneous injections and are generally safe and well-tolerated at different doses. In animal studies, combination of Pozelimab with Cemdisiran allowed lower doses and decreased dosing frequency compared to use of the individual agents separately [86]. The phase 3 randomised controlled trial of the combination (intravenous Pozelimab loading followed by 4-weekly subcutaneous injections along with Cemdisiran 200 mg subcutaneous 4-weekly) versus placebo in gMG is ongoing (NCT05070858).

### FcRn inhibitors

Neonatal Fc receptors (FcRn) are widely distributed in various cells, particularly endothelial and myeloid cells. FcRn helps in the recycling of IgG and increases its longevity in circulation. The binding of FcRn to IgG is pH dependent and occurs only in an acidic pH. After the uptake of IgG by the cells, it is transported to the acidic environment of endosomes where FcRn binds to IgG and prevents it from degraded by the lysosomal enzymes. FcRn transports IgG back to the surface and releases it in the neutral physiological pH [87]. This pathway prolongs the half-life of IgG antibodies which typically remain in circulation nearly 4 times longer than the other immunoglobulin subtypes like IgM and IgA. Serum albumin shares the long half-life and FcRn-mediated recycling by binding to a site distinct from that of IgG [88]. Serum proteins and IgG molecules which do not bind to FcRn are degraded within the lysosomes [89].

Blocking of FcRn can interfere with recycling of IgG antibodies associated with MG and many authors consider this technique similar to a “medical plasma exchange”, given the rapid reduction in IgG levels. While reducing the autoreactive IgG antibodies, these agents keep the concentrations of IgM and IgA antibodies stable [90]. A proportion (~25%) of the normal IgG response is retained and recovery of IgG levels occur faster compared to B cell depleting therapies [91]. Hence these therapies mount an effective immune response without increasing the risk of infections.

The various molecules studied in MG include engineered Fc fragments (Efgartigimod), mAb against FcRn (Rozanolixizumab, Nipocalimab, Batoclimab, and Orilanolimab), and peptide fragments.

#### Efgartigimod

Efgartigimod is an engineered Fc domain of human IgG1 with increased affinity for FcRn receptors than the endogenous IgG, and thus competitively inhibits IgG recycling [89]. The phase 3 multicentric randomised controlled ADAPT trial of efgartigimod studied 167 patients and proved its efficacy against placebo as an add-on therapy for generalised MG. Compared to placebo, Efgartigimod treated AChR-MG had significant improvement in MG-ADL score at 4 weeks (primary outcome). The other efficacy scores (QMG, MGC and MGQoL15 revised) showed a similar pattern with the maximum improvement noted by 4–5 weeks and sustained for 7 weeks. In total, 40–70% reduction in total IgG and AChR antibody levels were noted within the first week of the initial dose, recovering by 12 weeks and the levels corresponded inversely with clinical improvement. The drug was tolerated well and adverse effects including serious ones were no more common than in placebo [92].

#### Nipocalimab

Nipocalimab is a fully human aglycosylated IgG1 mAb against FcRn which strongly binds to FcRn independent of pH. In the phase 2 study (NCT03772587), 68 patients with gMG were found to have dose dependent reduction in IgG and anti-AChR antibody levels which correlated with clinical improvement [93]. Results are awaited from the ongoing phase 3 randomised controlled study in adults (NCT04951622) and phase 2/3 study open-label study in children (NCT05265273).

#### Rozanolixizumab

Rozanolixizumab is a high affinity humanised IgG4 mAb against FcRn which is administered subcutaneously. This drug completed phase 2 trial in 43 patients with moderate to severe gMG who were positive for either of AChR or MuSK antibodies. The primary efficacy outcome of improvement in QMG score from day 1 to 29 was not significant but overall data including antibody measurements suggested potential efficacy for the drug in moderate to severe gMG, without any significant side effects [94]. The phase 3 study (NCT03971422) is ongoing.

### B cell and plasma cell depleting therapies

In the presence of follicular dendritic cells, pro-inflammatory T helper cells and cytokines, there is proliferation of the autoreactive B cells within the thymic germinal centres and in the periphery, driving the pathogenesis of MG. The autoreactive B cells differentiate into antibody secreting plasma cells [95, 96].

In MG the B cells can be directly depleted or inhibited or indirectly by targeting their facilitators like cytokines or other immune cells [97]. Rituximab, a chimeric mouse/human antiCD20 mAb is already a well-established treatment in MG, especially with MuSK antibodies (produced by short-living plasma cells) and has been described earlier.

#### Inebilizumab

Inebilizumab is a humanised mAb targeting CD19 which is expressed in a broader group of B lineage cells compared to CD20, especially on the early pro-B cells and the majority of plasma cells in blood and secondary lymphoid organs and about half of the plasma cells in the bone marrow [98]. Antibody dependent cell mediated cytotoxicity is the primary mechanism of action of Inebilizumab which is currently undergoing phase 3 clinical studies in MG (NCT04524273).

#### Bortezomib

Bortezomib, targets plasma cells by a potent and reversible proteasome inhibition. Proteasomes are ubiquitous intracellular protein complexes and play a central role in protein homoeostasis by regulating the cell turnover and mediating the degradation of pro-apoptotic factors. As plasma cells are terminally differentiated and non-dividing, they are usually resistant to radiotherapy, glucocorticoids, standard oral immunosuppressants, and CD19/20 inhibitors. The rapid intracellular synthesis of antibodies in these cells render them highly susceptible to mechanisms which deter the protein degradation pathways like proteasome inhibitors. In addition, Bortezomib also targets nuclear factor κB (NF-κB) signalling pathway which has important anti-apoptotic functions and is often upregulated in inflammatory diseases.

Bortezomib, though primarily used for myeloma and mantle cell lymphoma, is a potential therapeutic option in a variety of refractory autoimmune diseases including MG, although the trial was terminated due to poor recruitment [99]. There are experimental data and case reports suggesting potential benefits in MG, but its use is limited by dose-dependent neurotoxicity in the form of polyneuropathy. The propensity for neuropathy is diminished by restricting the dose to a single cycle of bortezomib which appears sufficient to induce remission in autoimmune diseases and by using subcutaneous rather than intravenous route of administration [100].

#### Tolebrutinib

Bruton’s Tyrosine Kinase (BTK) belongs to the protein kinase family and is important in the signalling pathways involved in B cell proliferation and function including production of antibodies and cytokines, and antigen presentation. It is also expressed in myeloid cells (components of innate immunity) and influences the production of inflammatory cytokines and adhesion molecules which promote inflammation [101]. The suppression of these functions by BTK inhibitors like Tolebrutinib, results in their efficacy in autoimmune diseases and B cell malignancies. This orally administered irreversible covalent BTK inhibitor, is currently being evaluated in a phase 3 placebo-controlled randomised trial in MG (NCT05132569).

### Cytokine and chemokine targeting therapies

Cytokines are small signalling molecules which help in the coordination of immune system function and communication between the various immune and inflammatory cells. These are secreted by stimulated cells (chiefly T helper cells and macrophages) and act on a variety of target cells which harbour their specific receptors. Chemokines are a specific subgroup of cytokines with chemoattractant properties that attract leucocytes to sites of inflammation.94

The targeting of various cytokines have been attempted or is currently underway in MG, including Belimumab a human recombinant neutralising mAb against the B-cell activating factor (BAFF) [102, 103], Iscalimab a fully human non-depleting antiCD40 mAb which targets the activation and signalling pathways mediated by CD40 (which is a co-stimulatory molecule expressed in B cells and other antigen presenting cells which interacts with its ligand CD154 (CD40L) located on activated T cells, essential for the T cell dependent antibody responses, and the differentiation, survival and activation of memory B cells) [104] and IL-6 receptor antagonists like Tocilizumab and Satralizumab [105, 106].

### Chimeric autoantibody receptor (CAAR) T cell therapy

Chimeric antigen receptor (CAR) T cell therapy has shown marked success in the treatment of B cell malignancies and involves harvesting of T cells from the patient and genetically modifying them by attaching an artificially engineered receptor referred to as chimeric antigen receptor (CAR). When the CAR T cells are infused into the circulation, the modified receptors bind to a specific antigen on the target cells (e.g., cancerous cells in B cell malignancies) and destroy them. The effect of CAR T cells is sustained long-term by their in vivo multiplication [107]. This can be modified to be used in autoimmune diseases where a specific pathogenic antibody is identified, by using an engineered T cell which can bind the specific autoreactive B-cell. This technique is referred to as Chimeric autoantibody receptor (CAAR) T cell therapy and by specifically targeting B cells which express the autoantibody, a general depletion of the B cell lineage cells is avoided. The ongoing studies in MG include engineered T cells directed against B cell Maturation Antigen (BCMA) (NCT04146051) and MuSK antibody (NCT05451212).

### Stem cell transplantation

Autologous hematopoietic stem cell transplantation (HSCT) is being increasingly used for eliminating disease activity in various autoimmune neurological conditions including MG. The procedure involves stimulating the production of hematopoietic cells, harvesting them from circulation, ablating and resetting the immune system and then re-infusing the treated cells from the patient. The evidence for HSCT in MG is limited to a handful of patients [108]. The high risk for debilitating side effects including infections, secondary autoimmune diseases, and neoplasms makes HSCT a harder choice among the various treatment options, but nevertheless may need to be considered in selected refractory patients.

## Conclusions

**Fig. 4:**
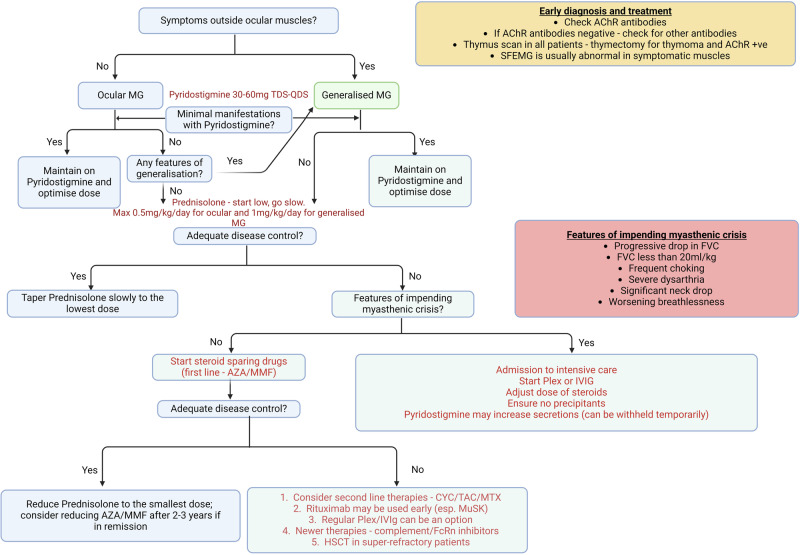
Diagnostic and treatment algorithm for myasthenia gravis. A simplified approach to the diagnosis and treatment of myasthenia gravis with clinical clues to look out for myasthenic crisis. AChR acetylcholine receptor, AZA Azathioprine, CYC Ciclosporin, FVC Forced Vital Capacity, HSCT haematopoietic stem cell transplantation, IVIG intravenous immunoglobulin, MG myasthenia gravis, MMF Mycophenolate mofetil, MTX Methotrexate, MuSK muscle specific tyrosine kinase, Plex plasma exchange, SFEMG single fibre electromyogram, TAC Tacrolimus.

A good proportion of patients with MG will present to the eye clinic with ptosis and double vision. It is important to be aware of the differential diagnosis and the bedside tests as well as neurophysiological and immunological tests which will aid the diagnosis. A stepwise approach to diagnosis and treatment with clinical clues to looks out for impending myasthenic crisis is shown in Fig. 4. Traditional treatments with cholinesterase inhibitors, corticosteroids and steroid sparing immunosuppressants are still the mainstay in the management of vast majority of patients with MG. However, ophthalmologists would need to be aware of the vast array of newer treatments which are being used in MG and where possible, work in collaboration with specialist neuroimmunology clinics.
